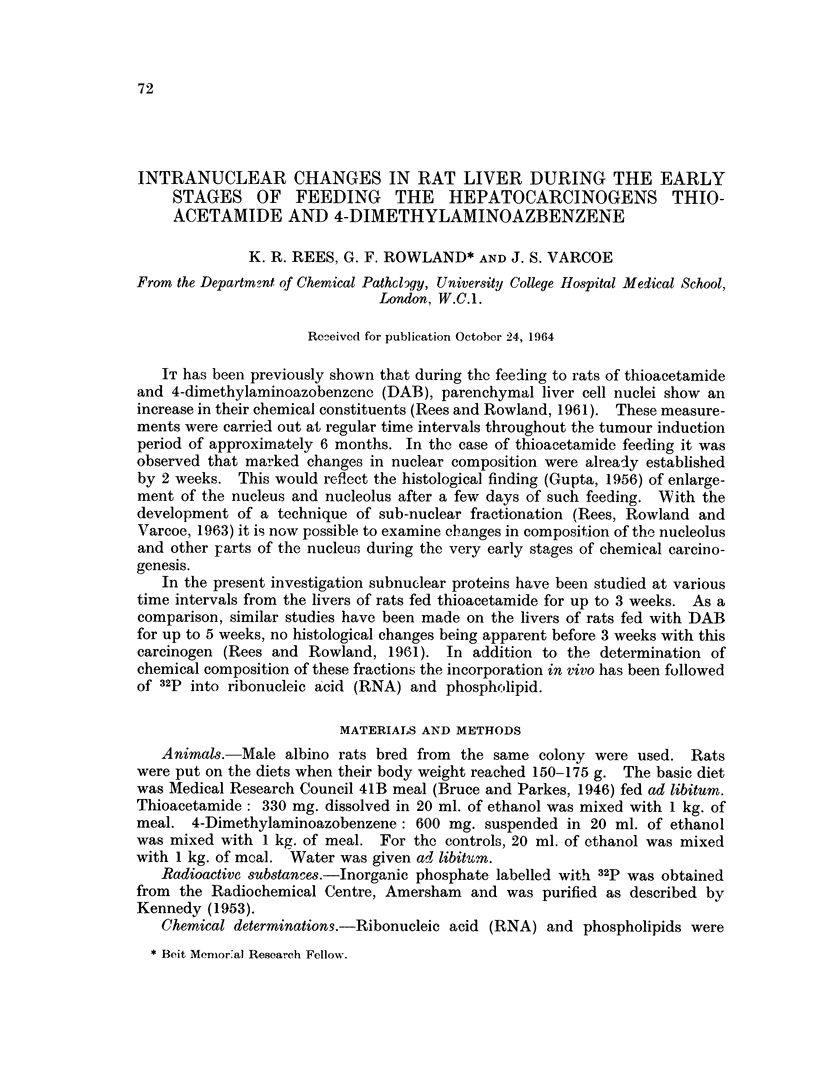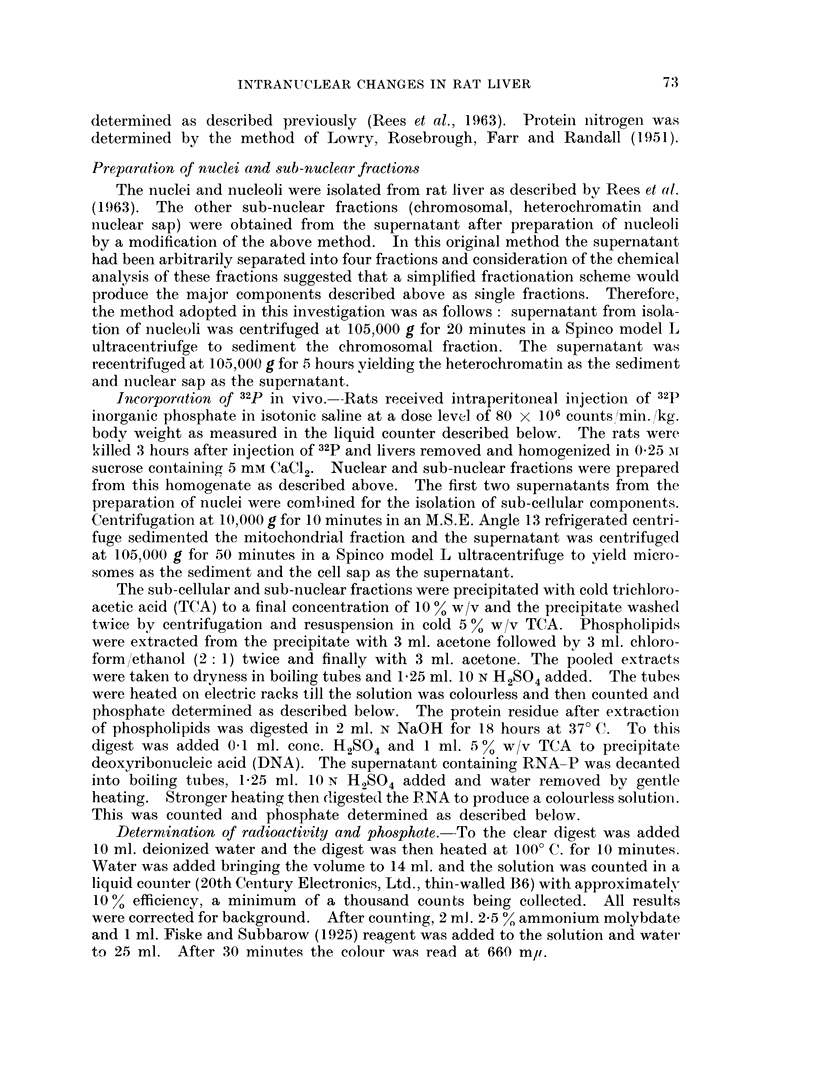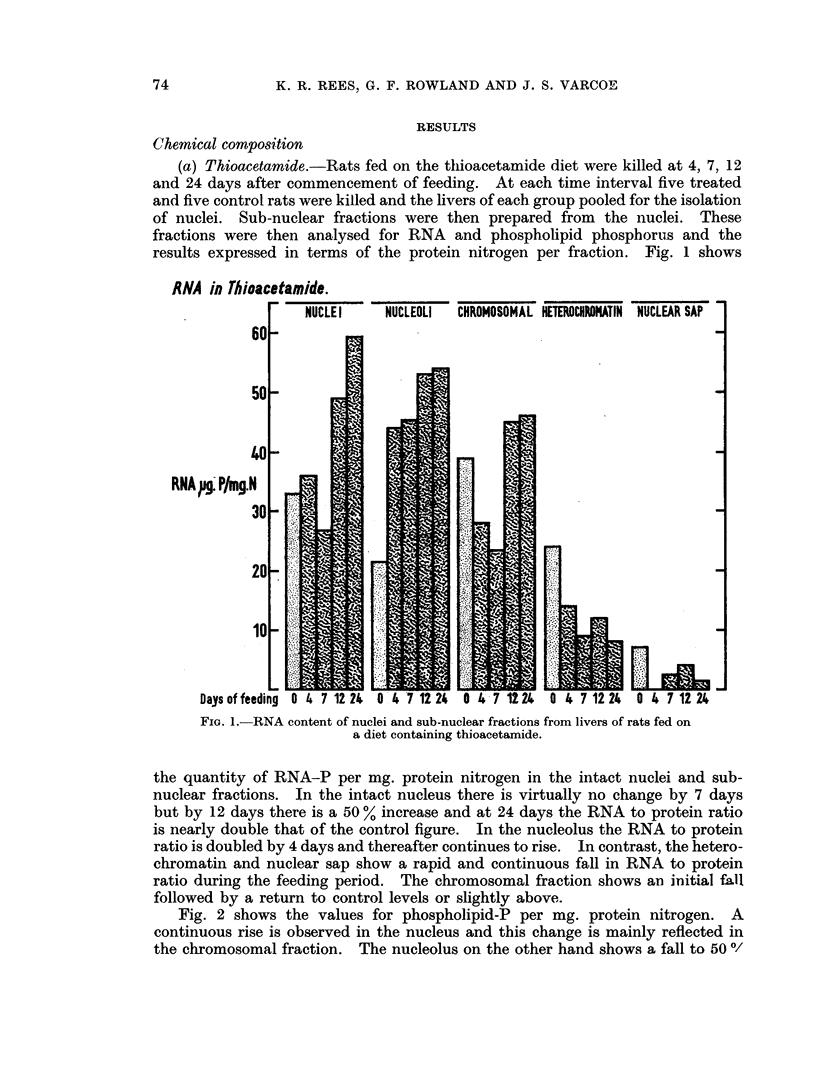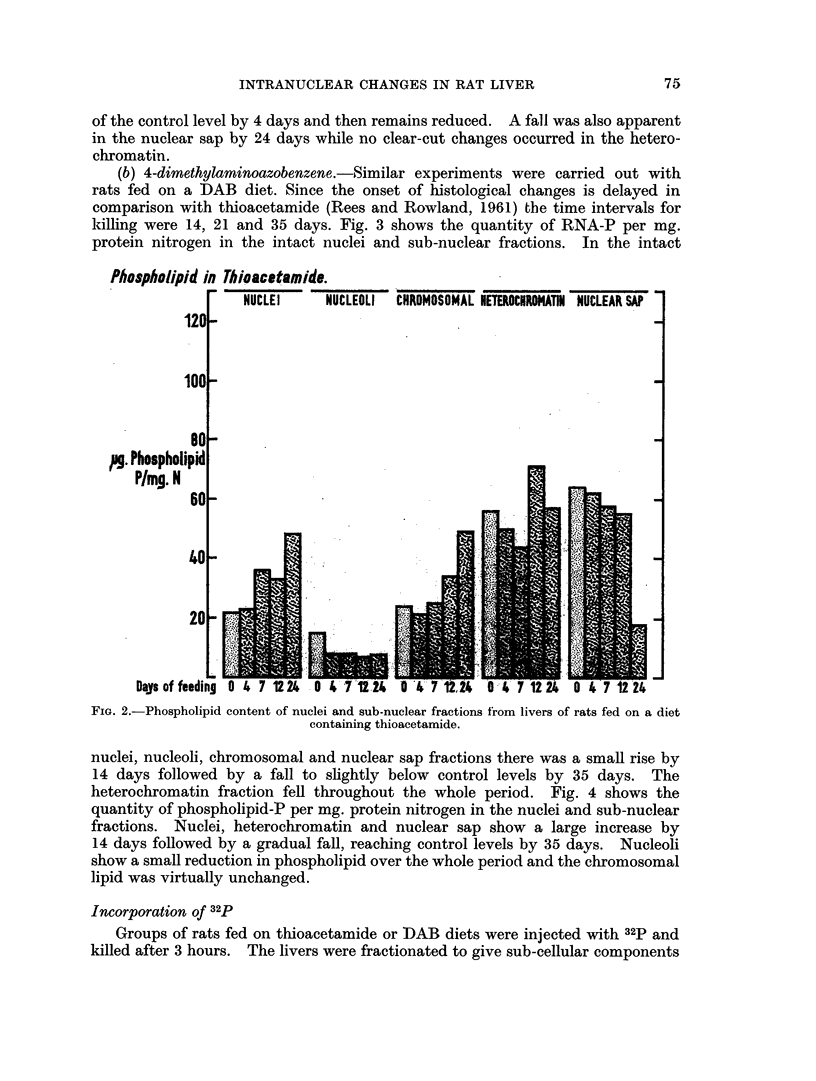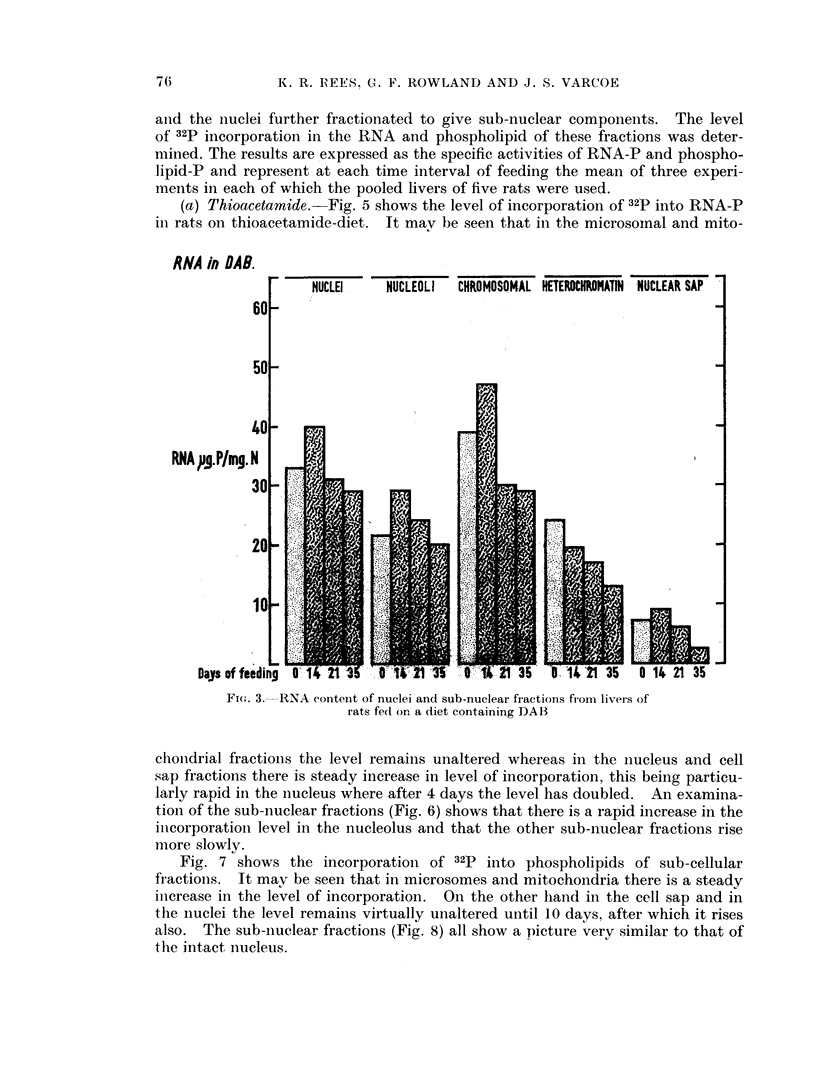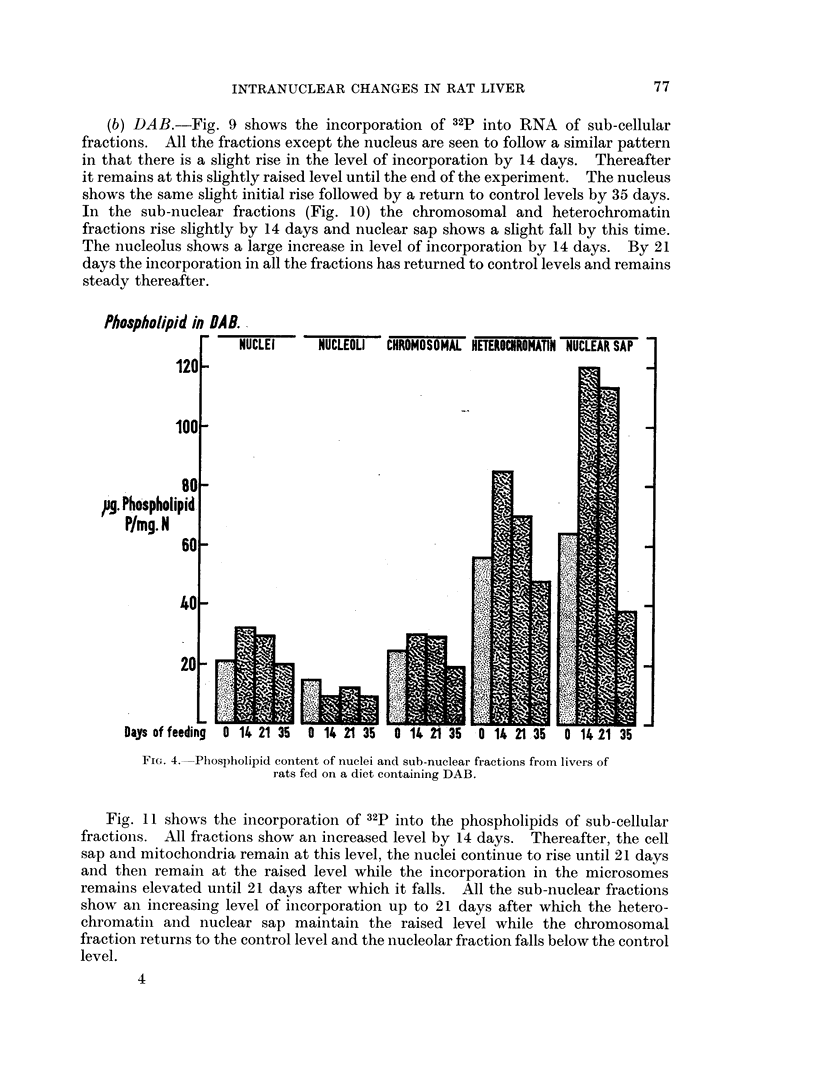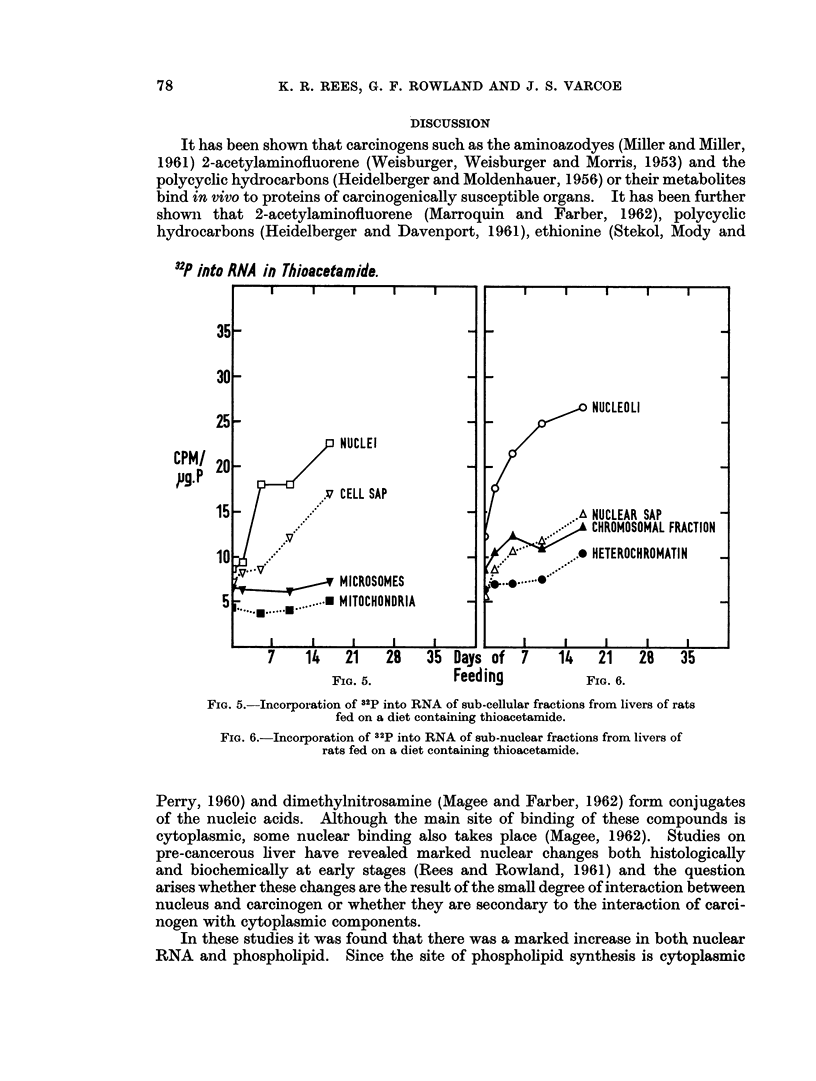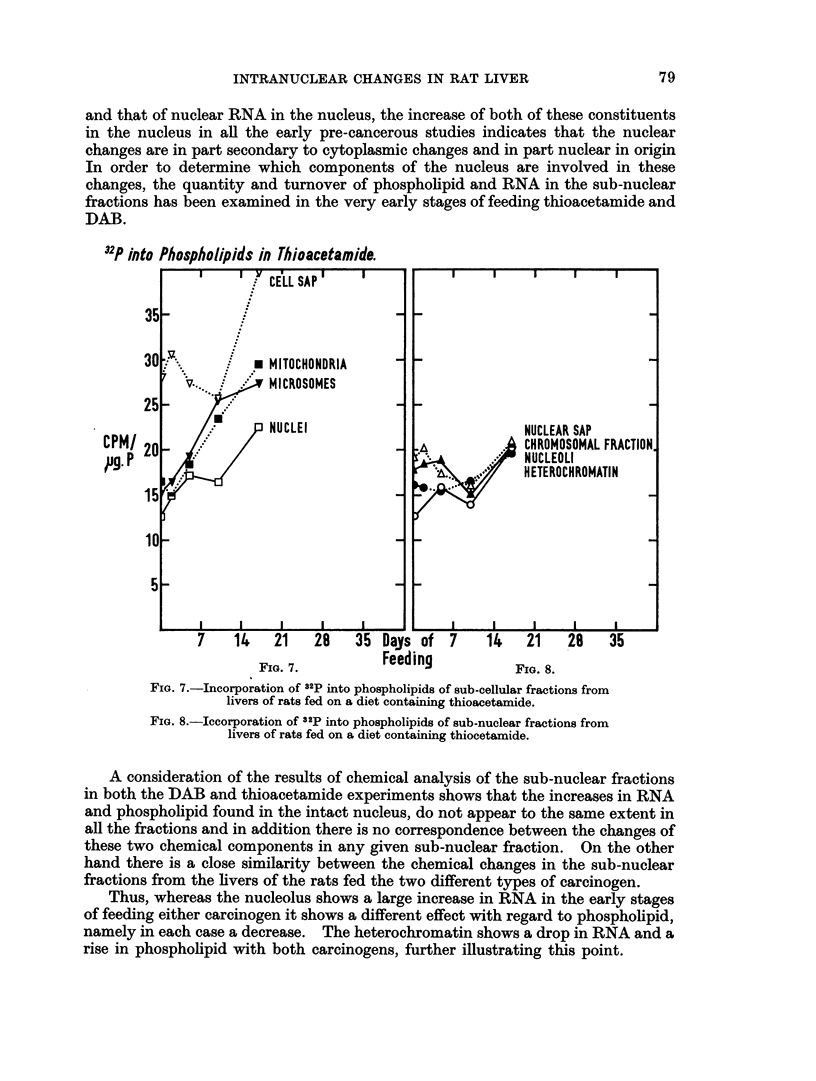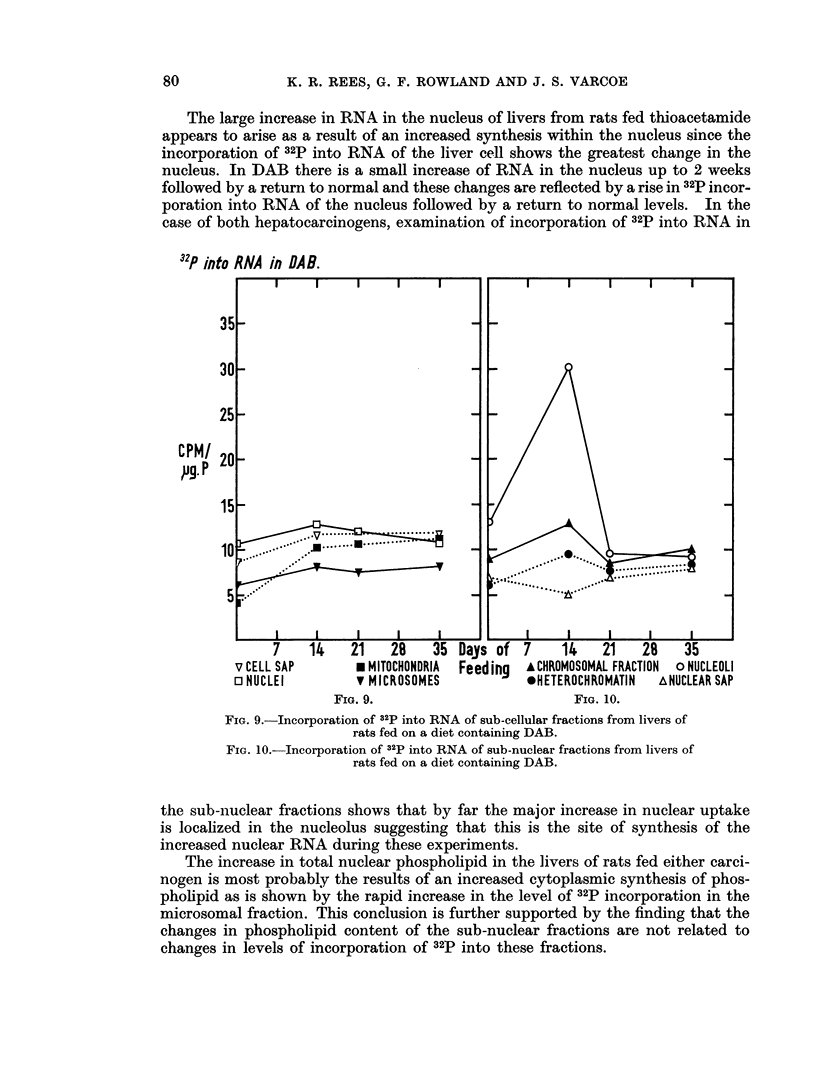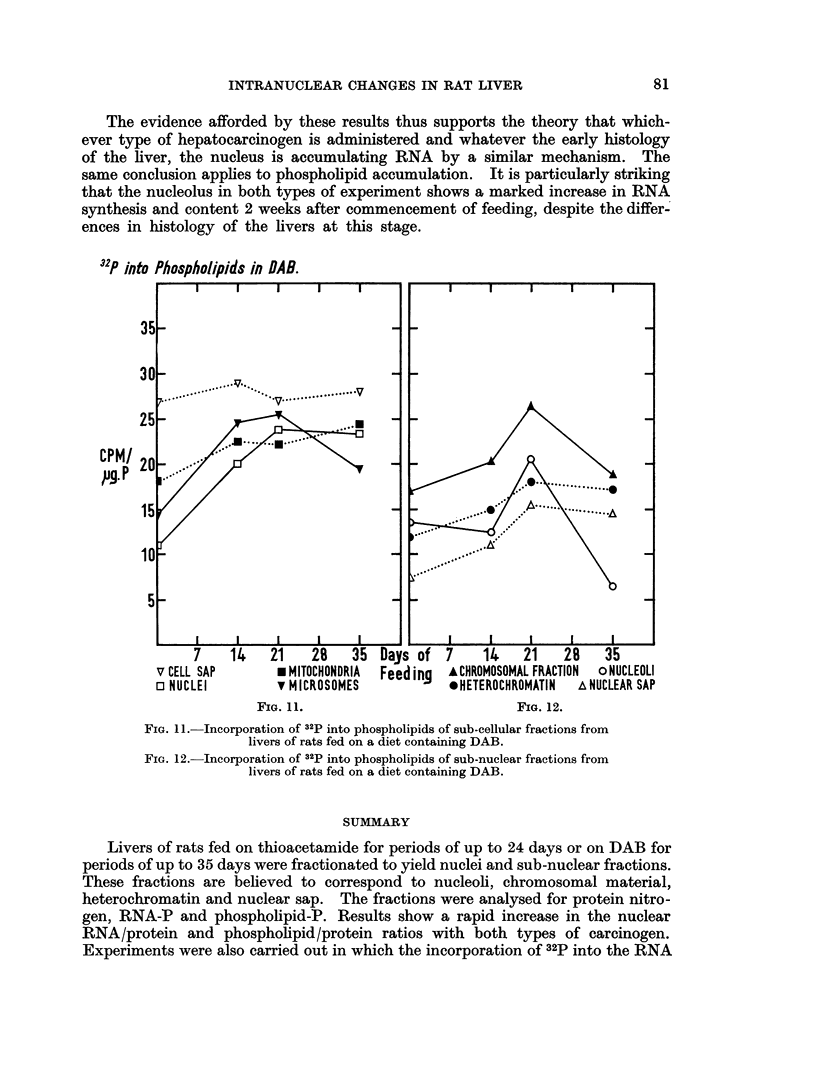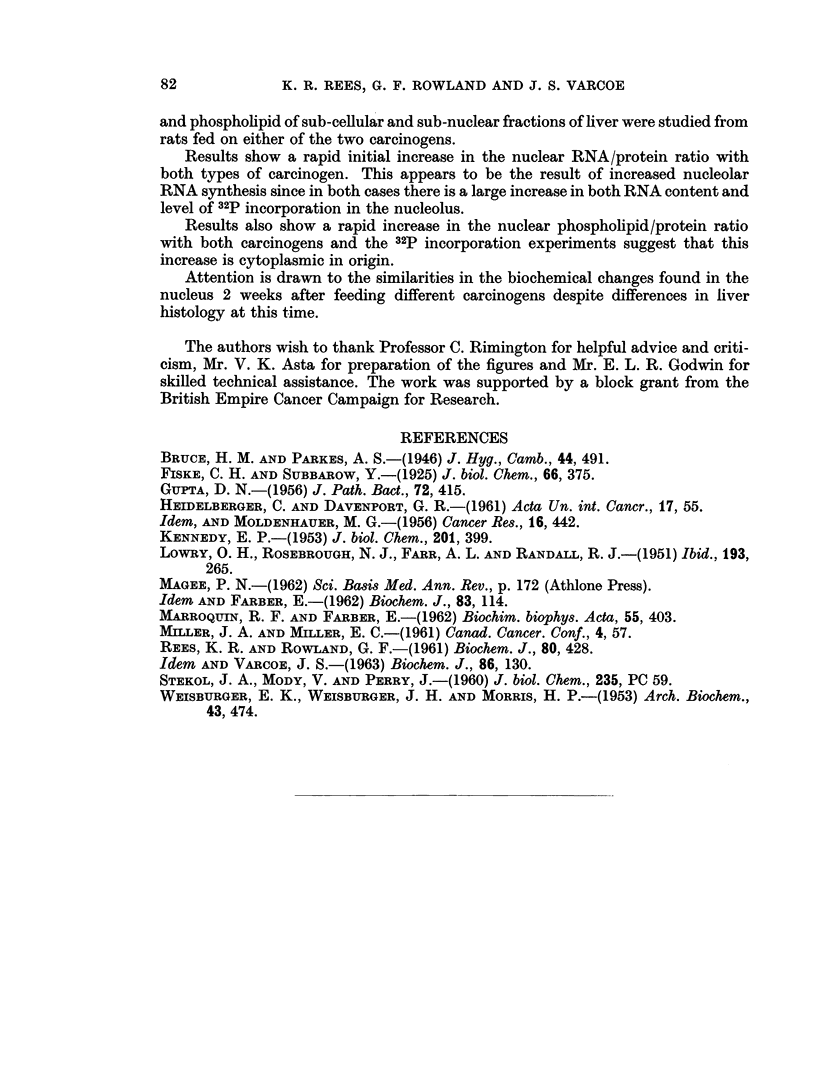# Intranuclear Changes in Rat Liver During the Early Stages of Feeding the Hepatocarcinogens Thioacetamide and 4-Dimethylaminoazobenzene

**DOI:** 10.1038/bjc.1965.7

**Published:** 1965-03

**Authors:** K. R. Rees, G. F. Rowland, J. S. Varcoe


### INTRANUCLEAR CHANGES IN RAT LIVER DURING THE EARLY STAGES OF FEEDING THE HEPATOCARCINOGENS THIOACETAMIDE AND 4-DIMETHYLAMINOAZBENZENE

K. R. REES, G. F. ROWLAND* AND J. S. VARCOE

*From the Department of Chemical Pathology, University College Hospital Medical School, London, W.C.1.*

Received for publication October 24, 1964

IT has been previously shown that during the feeding to rats of thioacetamide and 4-dimethylaminoazobenzene (DAB), parenchymal liver cell nuclei show an increase in their chemical constituents (Rees and Rowland, 1961). These measurements were carried out at regular time intervals throughout the tumour induction period of approximately 6 months. In the case of thioacetamide feeding it was observed that marked changes in nuclear composition were already established by 2 weeks. This would reflect the histological finding (Gupta, 1956) of enlargement of the nucleus and nucleolus after a few days of such feeding. With the development of a technique of sub-nuclear fractionation (Rees, Rowland and Varcoe, 1963) it is now possible to examine changes in composition of the nucleolus and other parts of the nucleus during the very early stages of chemical carcinogenesis.

In the present investigation subnuclear proteins have been studied at various time intervals from the livers of rats fed thioacetamide for up to 3 weeks. As a comparison, similar studies have been made on the livers of rats fed with DAB for up to 5 weeks, no histological changes being apparent before 3 weeks with this carcinogen (Rees and Rowland, 1961). In addition to the determination of chemical composition of these fractions the incorporation *in vivo* has been followed of $^{32}P$ into ribonucleic acid (RNA) and phospholipid.

### MATERIALS AND METHODS

*Animals.*—Male albino rats bred from the same colony were used. Rats were put on the diets when their body weight reached 150–175 g. The basic diet was Medical Research Council 41B meal (Bruce and Parkes, 1946) fed *ad libitum.* Thioacetamide : 330 mg. dissolved in 20 ml. of ethanol was mixed with 1 kg. of meal. 4-Dimethylaminoazobenzene : 600 mg. suspended in 20 ml. of ethanol was mixed with 1 kg. of meal. For the controls, 20 ml. of ethanol was mixed with 1 kg. of meal. Water was given *ad libitum.*

*Radioactive substances.*—Inorganic phosphate labelled with $^{32}P$ was obtained from the Radiochemical Centre, Amersham and was purified as described by Kennedy (1953).

*Chemical determinations.*—Ribonucleic acid (RNA) and phospholipids were

* Beit Memorial Research Fellow.

determined as described previously (Rees *et al.*, 1963). Protein nitrogen was determined by the method of Lowry, Rosebrough, Farr and Randall (1951).

*Preparation of nuclei and sub-nuclear fractions*

The nuclei and nucleoli were isolated from rat liver as described by Rees *et al.* (1963). The other sub-nuclear fractions (chromosomal, heterochromatin and nuclear sap) were obtained from the supernatant after preparation of nucleoli by a modification of the above method. In this original method the supernatant had been arbitrarily separated into four fractions and consideration of the chemical analysis of these fractions suggested that a simplified fractionation scheme would produce the major components described above as single fractions. Therefore, the method adopted in this investigation was as follows : supernatant from isolation of nucleoli was centrifuged at 105,000 ***g*** for 20 minutes in a Spinco model L ultracentriufge to sediment the chromosomal fraction. The supernatant was recentrifuged at 105,000 ***g*** for 5 hours yielding the heterochromatin as the sediment and nuclear sap as the supernatant.

*Incorporation of $^{32}P$* in vivo.—Rats received intraperitoneal injection of $^{32}P$ inorganic phosphate in isotonic saline at a dose level of $80 \times 10^6$ counts/min./kg. body weight as measured in the liquid counter described below. The rats were killed 3 hours after injection of $^{32}P$ and livers removed and homogenized in 0·25 M sucrose containing 5 mM $CaCl_2$. Nuclear and sub-nuclear fractions were prepared from this homogenate as described above. The first two supernatants from the preparation of nuclei were combined for the isolation of sub-cellular components. Centrifugation at 10,000 ***g*** for 10 minutes in an M.S.E. Angle 13 refrigerated centrifuge sedimented the mitochondrial fraction and the supernatant was centrifuged at 105,000 ***g*** for 50 minutes in a Spinco model L ultracentrifuge to yield microsomes as the sediment and the cell sap as the supernatant.

The sub-cellular and sub-nuclear fractions were precipitated with cold trichloroacetic acid (TCA) to a final concentration of 10 % w/v and the precipitate washed twice by centrifugation and resuspension in cold 5 % w/v TCA. Phospholipids were extracted from the precipitate with 3 ml. acetone followed by 3 ml. chloroform/ethanol (2 : 1) twice and finally with 3 ml. acetone. The pooled extracts were taken to dryness in boiling tubes and 1·25 ml. 10 N $H_2SO_4$ added. The tubes were heated on electric racks till the solution was colourless and then counted and phosphate determined as described below. The protein residue after extraction of phospholipids was digested in 2 ml. N NaOH for 18 hours at 37° C. To this digest was added 0·1 ml. conc. $H_2SO_4$ and 1 ml. 5 % w/v TCA to precipitate deoxyribonucleic acid (DNA). The supernatant containing RNA-P was decanted into boiling tubes, 1·25 ml. 10 N $H_2SO_4$ added and water removed by gentle heating. Stronger heating then digested the RNA to produce a colourless solution. This was counted and phosphate determined as described below.

*Determination of radioactivity and phosphate.*—To the clear digest was added 10 ml. deionized water and the digest was then heated at 100° C. for 10 minutes. Water was added bringing the volume to 14 ml. and the solution was counted in a liquid counter (20th Century Electronics, Ltd., thin-walled B6) with approximately 10 % efficiency, a minimum of a thousand counts being collected. All results were corrected for background. After counting, 2 ml. 2·5 % ammonium molybdate and 1 ml. Fiske and Subbarow (1925) reagent was added to the solution and water to 25 ml. After 30 minutes the colour was read at 660 m$\mu$.

### RESULTS

### *Chemical composition*

(*a*) *Thioacetamide.*—Rats fed on the thioacetamide diet were killed at 4, 7, 12 and 24 days after commencement of feeding. At each time interval five treated and five control rats were killed and the livers of each group pooled for the isolation of nuclei. Sub-nuclear fractions were then prepared from the nuclei. These fractions were then analysed for RNA and phospholipid phosphorus and the results expressed in terms of the protein nitrogen per fraction. Fig. 1 shows

FIG. 1.—RNA content of nuclei and sub-nuclear fractions from livers of rats fed on a diet containing thioacetamide.

the quantity of RNA–P per mg. protein nitrogen in the intact nuclei and sub-nuclear fractions. In the intact nucleus there is virtually no change by 7 days but by 12 days there is a 50 % increase and at 24 days the RNA to protein ratio is nearly double that of the control figure. In the nucleolus the RNA to protein ratio is doubled by 4 days and thereafter continues to rise. In contrast, the heterochromatin and nuclear sap show a rapid and continuous fall in RNA to protein ratio during the feeding period. The chromosomal fraction shows an initial fall followed by a return to control levels or slightly above.

Fig. 2 shows the values for phospholipid-P per mg. protein nitrogen. A continuous rise is observed in the nucleus and this change is mainly reflected in the chromosomal fraction. The nucleolus on the other hand shows a fall to 50 %

of the control level by 4 days and then remains reduced. A fall was also apparent in the nuclear sap by 24 days while no clear-cut changes occurred in the heterochromatin.

(*b*) *4-dimethylaminoazobenzene*.—Similar experiments were carried out with rats fed on a DAB diet. Since the onset of histological changes is delayed in comparison with thioacetamide (Rees and Rowland, 1961) the time intervals for killing were 14, 21 and 35 days. Fig. 3 shows the quantity of RNA-P per mg. protein nitrogen in the intact nuclei and sub-nuclear fractions. In the intact

FIG. 2.—Phospholipid content of nuclei and sub-nuclear fractions from livers of rats fed on a diet containing thioacetamide.

nuclei, nucleoli, chromosomal and nuclear sap fractions there was a small rise by 14 days followed by a fall to slightly below control levels by 35 days. The heterochromatin fraction fell throughout the whole period. Fig. 4 shows the quantity of phospholipid-P per mg. protein nitrogen in the nuclei and sub-nuclear fractions. Nuclei, heterochromatin and nuclear sap show a large increase by 14 days followed by a gradual fall, reaching control levels by 35 days. Nucleoli show a small reduction in phospholipid over the whole period and the chromosomal lipid was virtually unchanged.

*Incorporation of* $^{32}P$

Groups of rats fed on thioacetamide or DAB diets were injected with $^{32}P$ and killed after 3 hours. The livers were fractionated to give sub-cellular components and the nuclei further fractionated to give sub-nuclear components. The level of $^{32}P$ incorporation in the RNA and phospholipid of these fractions was determined. The results are expressed as the specific activities of RNA-P and phospholipid-P and represent at each time interval of feeding the mean of three experiments in each of which the pooled livers of five rats were used.

(*a*) *Thioacetamide.*—Fig. 5 shows the level of incorporation of $^{32}P$ into RNA-P in rats on thioacetamide-diet. It may be seen that in the microsomal and mito-

FIG. 3.—RNA content of nuclei and sub-nuclear fractions from livers of rats fed on a diet containing DAB

chondrial fractions the level remains unaltered whereas in the nucleus and cell sap fractions there is steady increase in level of incorporation, this being particularly rapid in the nucleus where after 4 days the level has doubled. An examination of the sub-nuclear fractions (Fig. 6) shows that there is a rapid increase in the incorporation level in the nucleolus and that the other sub-nuclear fractions rise more slowly.

Fig. 7 shows the incorporation of $^{32}P$ into phospholipids of sub-cellular fractions. It may be seen that in microsomes and mitochondria there is a steady increase in the level of incorporation. On the other hand in the cell sap and in the nuclei the level remains virtually unaltered until 10 days, after which it rises also. The sub-nuclear fractions (Fig. 8) all show a picture very similar to that of the intact nucleus.

(*b*) *DAB*.—Fig. 9 shows the incorporation of $^{32}P$ into RNA of sub-cellular fractions. All the fractions except the nucleus are seen to follow a similar pattern in that there is a slight rise in the level of incorporation by 14 days. Thereafter it remains at this slightly raised level until the end of the experiment. The nucleus shows the same slight initial rise followed by a return to control levels by 35 days. In the sub-nuclear fractions (Fig. 10) the chromosomal and heterochromatin fractions rise slightly by 14 days and nuclear sap shows a slight fall by this time. The nucleolus shows a large increase in level of incorporation by 14 days. By 21 days the incorporation in all the fractions has returned to control levels and remains steady thereafter.

FIG. 4.—Phospholipid content of nuclei and sub-nuclear fractions from livers of rats fed on a diet containing DAB.

Fig. 11 shows the incorporation of $^{32}P$ into the phospholipids of sub-cellular fractions. All fractions show an increased level by 14 days. Thereafter, the cell sap and mitochondria remain at this level, the nuclei continue to rise until 21 days and then remain at the raised level while the incorporation in the microsomes remains elevated until 21 days after which it falls. All the sub-nuclear fractions show an increasing level of incorporation up to 21 days after which the heterochromatin and nuclear sap maintain the raised level while the chromosomal fraction returns to the control level and the nucleolar fraction falls below the control level.

### DISCUSSION

It has been shown that carcinogens such as the aminoazodyes (Miller and Miller, 1961) 2-acetylaminofluorene (Weisburger, Weisburger and Morris, 1953) and the polycyclic hydrocarbons (Heidelberger and Moldenhauer, 1956) or their metabolites bind *in vivo* to proteins of carcinogenically susceptible organs. It has been further shown that 2-acetylaminofluorene (Marroquin and Farber, 1962), polycyclic hydrocarbons (Heidelberger and Davenport, 1961), ethionine (Stekol, Mody and

Fig. 5. Fig. 6.

Fig. 5.—Incorporation of $^{32}P$ into RNA of sub-cellular fractions from livers of rats fed on a diet containing thioacetamide.

Fig. 6.—Incorporation of $^{32}P$ into RNA of sub-nuclear fractions from livers of rats fed on a diet containing thioacetamide.

Perry, 1960) and dimethylnitrosamine (Magee and Farber, 1962) form conjugates of the nucleic acids. Although the main site of binding of these compounds is cytoplasmic, some nuclear binding also takes place (Magee, 1962). Studies on pre-cancerous liver have revealed marked nuclear changes both histologically and biochemically at early stages (Rees and Rowland, 1961) and the question arises whether these changes are the result of the small degree of interaction between nucleus and carcinogen or whether they are secondary to the interaction of carcinogen with cytoplasmic components.

In these studies it was found that there was a marked increase in both nuclear RNA and phospholipid. Since the site of phospholipid synthesis is cytoplasmic and that of nuclear RNA in the nucleus, the increase of both of these constituents in the nucleus in all the early pre-cancerous studies indicates that the nuclear changes are in part secondary to cytoplasmic changes and in part nuclear in origin In order to determine which components of the nucleus are involved in these changes, the quantity and turnover of phospholipid and RNA in the sub-nuclear fractions has been examined in the very early stages of feeding thioacetamide and DAB.

Fig. 7. Fig. 8.

Fig. 7.—Incorporation of $^{32}P$ into phospholipids of sub-cellular fractions from livers of rats fed on a diet containing thioacetamide.

Fig. 8.—Iccorporation of $^{32}P$ into phospholipids of sub-nuclear fractions from livers of rats fed on a diet containing thiocetamide.

A consideration of the results of chemical analysis of the sub-nuclear fractions in both the DAB and thioacetamide experiments shows that the increases in RNA and phospholipid found in the intact nucleus, do not appear to the same extent in all the fractions and in addition there is no correspondence between the changes of these two chemical components in any given sub-nuclear fraction. On the other hand there is a close similarity between the chemical changes in the sub-nuclear fractions from the livers of the rats fed the two different types of carcinogen.

Thus, whereas the nucleolus shows a large increase in RNA in the early stages of feeding either carcinogen it shows a different effect with regard to phospholipid, namely in each case a decrease. The heterochromatin shows a drop in RNA and a rise in phospholipid with both carcinogens, further illustrating this point.

The large increase in RNA in the nucleus of livers from rats fed thioacetamide appears to arise as a result of an increased synthesis within the nucleus since the incorporation of $^{32}P$ into RNA of the liver cell shows the greatest change in the nucleus. In DAB there is a small increase of RNA in the nucleus up to 2 weeks followed by a return to normal and these changes are reflected by a rise in $^{32}P$ incorporation into RNA of the nucleus followed by a return to normal levels. In the case of both hepatocarcinogens, examination of incorporation of $^{32}P$ into RNA in

Fig. 9. Fig. 10.

Fig. 9.—Incorporation of $^{32}P$ into RNA of sub-cellular fractions from livers of rats fed on a diet containing DAB.

Fig. 10.—Incorporation of $^{32}P$ into RNA of sub-nuclear fractions from livers of rats fed on a diet containing DAB.

the sub-nuclear fractions shows that by far the major increase in nuclear uptake is localized in the nucleolus suggesting that this is the site of synthesis of the increased nuclear RNA during these experiments.

The increase in total nuclear phospholipid in the livers of rats fed either carcinogen is most probably the results of an increased cytoplasmic synthesis of phospholipid as is shown by the rapid increase in the level of $^{32}P$ incorporation in the microsomal fraction. This conclusion is further supported by the finding that the changes in phospholipid content of the sub-nuclear fractions are not related to changes in levels of incorporation of $^{32}P$ into these fractions.

The evidence afforded by these results thus supports the theory that whichever type of hepatocarcinogen is administered and whatever the early histology of the liver, the nucleus is accumulating RNA by a similar mechanism. The same conclusion applies to phospholipid accumulation. It is particularly striking that the nucleolus in both types of experiment shows a marked increase in RNA synthesis and content 2 weeks after commencement of feeding, despite the differences in histology of the livers at this stage.

FIG. 11. FIG. 12.

FIG. 11.—Incorporation of $^{32}P$ into phospholipids of sub-cellular fractions from livers of rats fed on a diet containing DAB.

FIG. 12.—Incorporation of $^{32}P$ into phospholipids of sub-nuclear fractions from livers of rats fed on a diet containing DAB.

### SUMMARY

Livers of rats fed on thioacetamide for periods of up to 24 days or on DAB for periods of up to 35 days were fractionated to yield nuclei and sub-nuclear fractions. These fractions are believed to correspond to nucleoli, chromosomal material, heterochromatin and nuclear sap. The fractions were analysed for protein nitrogen, RNA-P and phospholipid-P. Results show a rapid increase in the nuclear RNA/protein and phospholipid/protein ratios with both types of carcinogen. Experiments were also carried out in which the incorporation of $^{32}P$ into the RNA

and phospholipid of sub-cellular and sub-nuclear fractions of liver were studied from rats fed on either of the two carcinogens.

Results show a rapid initial increase in the nuclear RNA/protein ratio with both types of carcinogen. This appears to be the result of increased nucleolar RNA synthesis since in both cases there is a large increase in both RNA content and level of $^{32}P$ incorporation in the nucleolus.

Results also show a rapid increase in the nuclear phospholipid/protein ratio with both carcinogens and the $^{32}P$ incorporation experiments suggest that this increase is cytoplasmic in origin.

Attention is drawn to the similarities in the biochemical changes found in the nucleus 2 weeks after feeding different carcinogens despite differences in liver histology at this time.

The authors wish to thank Professor C. Rimington for helpful advice and criticism, Mr. V. K. Asta for preparation of the figures and Mr. E. L. R. Godwin for skilled technical assistance. The work was supported by a block grant from the British Empire Cancer Campaign for Research.

### REFERENCES

Bruce, H. M. and Parkes, A. S.—(1946) *J. Hyg., Camb.*, **44**, 491.
Fiske, C. H. and Subbarow, Y.—(1925) *J. biol. Chem.*, **66**, 375.
Gupta, D. N.—(1956) *J. Path. Bact.*, **72**, 415.
Heidelberger, C. and Davenport, G. R.—(1961) *Acta Un. int. Cancr.*, **17**, 55.
*Idem*, and Moldenhauer, M. G.—(1956) *Cancer Res.*, **16**, 442.
Kennedy, E. P.—(1953) *J. biol. Chem.*, **201**, 399.
Lowry, O. H., Rosebrough, N. J., Farr, A. L. and Randall, R. J.—(1951) *Ibid.*, **193**, 265.
Magee, P. N.—(1962) *Sci. Basis Med. Ann. Rev.*, p. 172 (Athlone Press).
*Idem* and Farber, E.—(1962) *Biochem. J.*, **83**, 114.
Marroquin, R. F. and Farber, E.—(1962) *Biochim. biophys. Acta*, **55**, 403.
Miller, J. A. and Miller, E. C.—(1961) *Canad. Cancer. Conf.*, **4**, 57.
Rees, K. R. and Rowland, G. F.—(1961) *Biochem. J.*, **80**, 428.
*Idem* and Varcoe, J. S.—(1963) *Biochem. J.*, **86**, 130.
Stekol, J. A., Mody, V. and Perry, J.—(1960) *J. biol. Chem.*, **235**, PC 59.
Weisburger, E. K., Weisburger, J. H. and Morris, H. P.—(1953) *Arch. Biochem.*, **43**, 474.